# Viscoelastic Properties of Polypropylene during Crystallization and Melting: Experimental and Phenomenological Modeling

**DOI:** 10.3390/polym15183846

**Published:** 2023-09-21

**Authors:** Noëlle Billon, Romain Castellani, Jean-Luc Bouvard, Guilhem Rival

**Affiliations:** 1 Mines Paris, PSL University, Centre for Material Forming (CEMEF), UMR CNRS 7635, 06904 Sophia Antipolis, France; romain.castellani@minesparis.psl.eu (R.C.); jean-luc.bouvard@minesparis.psl.eu (J.-L.B.); guilhem.rival@insa-lyon.fr (G.R.); 2 Lyon University, INSA-Lyon, LGEF, EA682, 69621 Villeurbanne, France

**Keywords:** crystallization, rheology, viscoelasticity

## Abstract

This paper deals with the viscoelastic behavior during crystallization and melting of semicrystalline polymers, with the aim of later modeling the residual stresses after processing in cases where crystallization occurs in quasi-static conditions (in additive manufacturing for example). Despite an abundant literature on polymer crystallization, the current state of scientific knowledge does not yet allow ab initio modeling. Therefore, an alternative and pragmatic way has been explored to propose a first approximation of the impact of crystallization and melting on the storage and loss moduli during crystallization–melting–crystallization cycles. An experimental approach, combining DSC, optical microscopy and oscillatory shear rheology, was used to define macroscopic parameters related to the microstructure. These parameters have been integrated into a phenomenological model. Isothermal measurements were used to describe the general framework, and crystallization at a constant cooling rate was used to evaluate the feasibility of a general approach. It can be concluded that relying solely on the crystalline fraction is inadequate to model the rheology. Instead, accounting for the microstructure at the spherulitic level could be more useful. Additionally, the results obtained from the experiments help to enhance our understanding of the correlations between crystallization kinetics and its mechanical effects.

## 1. Introduction

The general goal of this study is to progress in the understanding and the modeling of the linear viscoelasticity of polymers during their crystallization and melting stages. As a first step, it focuses on the case where no shear-induced crystallization occurs. The targeted applications are then welding or, even more importantly, the additive manufacturing of semi-crystalline polymers [1]. Unlike what has been widely studied in the past in the context of conventional processes, namely the effect of flow on crystallization kinetics [2], the question that arises in this study is how partial crystallization changes mechanical behavior to ultimately address the appearance of residual stresses and warpage [3].

Within this general frame, the main purpose of the present step is to propose a simple model to account for the effect of crystallization and melting on storage and loss moduli. To achieve that point, and following studies reported in the literature [4,5,6,7,8,9], investigations combining differential scanning calorimetry (DSC), optical microscopy (OM) and oscillatory rotational rheology were conducted in isothermal conditions and under constant cooling rates. A rheometer allowed the assessment of linear viscoelasticity during phase changes. DSC and OM allowed the assessment of crystallization (or melting) kinetics and growth rates, respectively.

Previously reported results did enable the drawing of general correlation between crystallization kinetics estimated from DSC analyses and rheological measurements. For example, the transformed volume fraction leading to a 10-fold viscosity increase reported in the literature varies from 2% to 40% [4,5,6,7,8,9]. Therefore, it is still worth performing experimental analysis, and some new inputs for models must be defined. Presumably, they should characterize the microstructure.

In consequence, based on classical overall crystallization theories, some macroscopic parameters, related to the microstructure, were suggested. They aimed at standing for the microstructure even though they cannot describe it precisely:

- The first is the transformed volume fraction, which is related to the amount of crystalline phase.
- The second is related to the number of spherulites.
- The third is related to the average radius of spherulites over time.
- The fourth is chosen to assess the level of impingement of spherulites.
- Finally, these parameters were correlated to the rheological measurements and a phenomenological model was proposed and validated. Therefore, storage and loss moduli could be reproduced during melting–crystallization cycles.

The paper first describes the theoretical framework, thereafter significant results are presented and finally the correlations between crystallization and rheological measurements are discussed. This significantly enriches general knowledge and elucidates the reason for the huge discrepancy between results in earlier literature.

In a last step, parameters are suggested and argued, and the model is developed and validated.

## 2. Materials and Methods

### 2.1. Materials

For this study, isotactic polypropylene (iPP) pellets supplied by Sigma Aldrich were used (Sigma-Aldrich Chimie, Saint Quentin Fallavier, France). The supplier indicates an average molecular weight ${\bar{M}}_{n}\approx 97\;\mathrm{Kg}\;{\mathrm{mol}}^{-1}$ and a mass average molecular weight ${\bar{M}}_{w}\approx 340\;\mathrm{Kg}\;{\mathrm{mol}}^{-1}$.

To carry out analyses, pellets were pressed into 1 mm thick disks. The protocol consisted of heating the pellets up to 220 °C in an aluminum mold placed in a hot press. After being at this temperature for 5 min, a pressure of approximately 0.7 MPa was applied on the mold and held for 5 more minutes. Finally, while keeping the pressure, the mold was cooled down to room temperature thanks to a water-cooling system. Accurate cooling rates inside the mold are not known. All the characterizations mentioned below were conducted with a step at elevated temperature to erase thermal history.

### 2.2. Differential Scanning Calorimetry

Differential scanning calorimetry (DSC) analyses were performed under nitrogen flow on a DSC 8500 manufactured by PerkinElmer (PerkinElmer France, Villebon-sur-Yvette, France). The sample weighed about 4 mg. These analyses were conducted to investigate crystallization kinetics under both non-isothermal and isothermal conditions.

**Figure A1:**
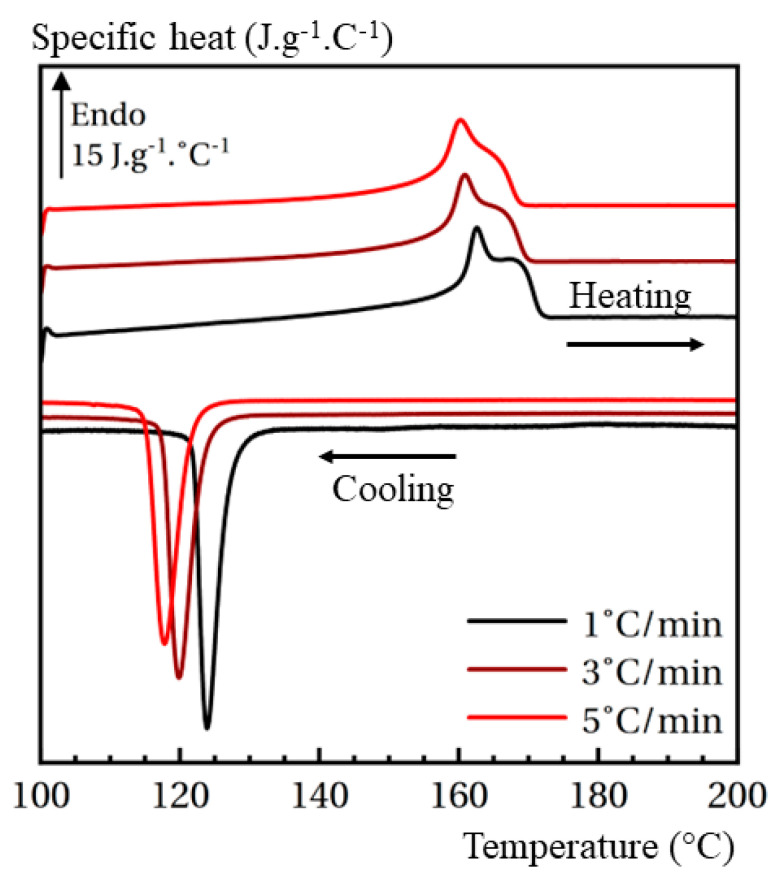
Normalized (heat flow/(mass. rate)) DSC heating–cooling cycles for three rates.

Under non-isothermal conditions, the sample was first heated to 200 °C to erase any previous thermal history. Then, the sample was cooled down to 100 °C at various rates (5 °C min^−1^, 3 °C min^−1^ and 1 °C min^−1^) and heated to 200 °C at the same rate. The three cycles were performed successively on the same sample (Figure A1 of Appendix A). First-order transition temperatures (melting temperature T_m_ and crystallization temperature T_c_) were measured at the maximum of the peaks. Crystallinity ratio, $X_{c}$, was deduced from the melting enthalpy, $\Delta H_{m}$, accounting for the theoretical melting enthalpy of a fully crystalline PP, $\Delta H_{\infty }$, Xc=ΔHmΔH∞×100.

Under isothermal conditions, the sample was also initially heated to 200 °C to erase thermal history. Then, the sample was cooled down at 20 °C min^−1^ to constant temperature. This latter was chosen to be well above the previously determined crystallization temperature for this rate. Then, the heat flow evolution was recorded as a function of time. This protocol was performed on the same sample for five isotherms: 125 °C, 127.5 °C, 130 °C, 132.5 °C and 135 °C.

### 2.3. Polarized Light Microscopy

**Figure A2:**
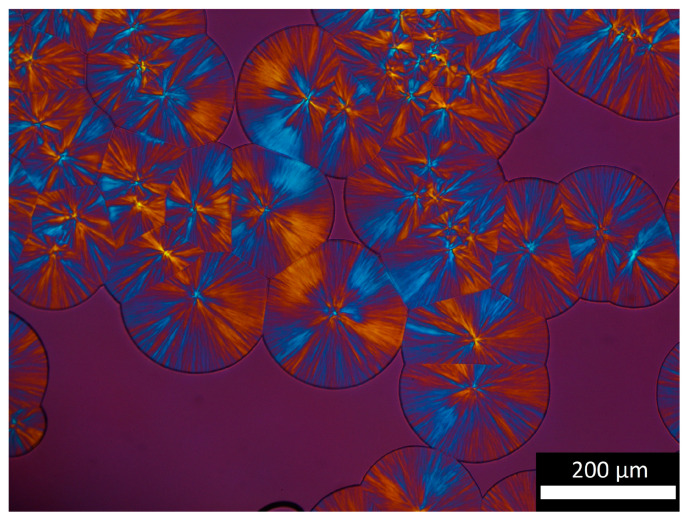
Typical image observed during isothermal crystallization. Temperature was 130 °C.

Polarized light microscopy observations were carried out at different isothermal temperatures to determine growth rate of iPP spherulites. A hot stage HS82 manufactured by Mettler Toledo (Mettler Toledo Viroflay, France) associated with a polarized light optical microscope (Leica DMRX, Leica Microsystemes, Nanteres, France) linked to a computer was used to perform growth rate analysis. A typical image is given in Appendix A (Figure A2). One can observe that nucleation was close to instantaneous as all spherulites had approximately the same radius.

To observe spherulite growth, the sample needs to be thin enough (typically a few hundreds of µm) to allow light transmission. To obtain such thin samples, a little piece was cut using a razor blade and then placed on a glass slide. Thereafter, the glass slide was heated, thanks to the heating sample holder, up to a temperature of 200 °C (above melting temperature). Once the sample melted, it was pressed under another glass slide to thin it.

**Figure A3:**
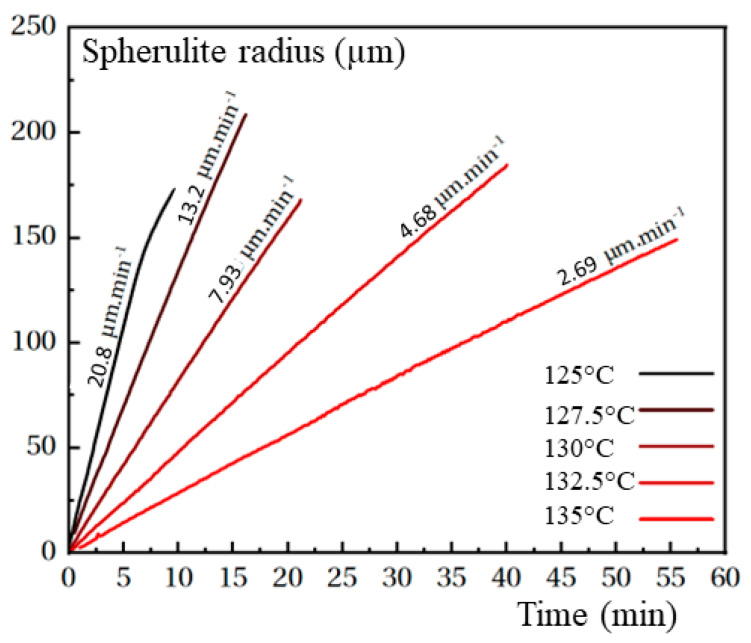
Radius of spherulites vs. time for the five crystallization temperatures. Figures on the curves are respective growth rates. Non-linearity seen at 125 °C was neglected.

Like isothermal calorimetric analyses, samples were then cooled at a rate of 20 °C min^−1^ to reach different isothermal temperatures (125 °C, 127.5 °C, 130 °C, 132.5 °C and 135 °C). Pictures of the sample were taken with a microscope at regular time intervals to record crystal growth. Analysis of each set of images (one for each temperature) was performed using ImageJ software. For each temperature, the radius of three spherulites was measured as a function of time and the growth rate was found by performing a linear fit. Results are depicted in Figure A3 of Appendix A.

### 2.4. Rheological Analyses

Rheological measurements focused on the complex shear modulus. Isothermal crystallization temperatures were high enough to ensure that crystallization was not started during the cooling step. In the case of non-isothermal measurements, the cooling rate was low to ensure that the temperature in the sample was still uniform. Consequently, the ranges of temperatures and rates are quite restricted, as in any equivalent study reported in the literature. For polypropylene, the polymer most often used as a model material, the range of crystallization temperatures lies between 145 °C and 130 °C. The cooling rate did not exceed a few degrees per minute.

In the present case, rheological analyses were carried out on an MCR-302 stress-controlled rheometer manufactured by Anton Paar (Anton Parr Courtaboeuf, Les Ullis, France), equipped with a CTD 450 Convection Temperature Control Device. Tests were conducted using 25 mm diameter parallel plate geometries with an initial gap height between plates of about 1 mm. To accommodate the changes in sample thickness, particularly during crystallization and melting phenomena where the polymer density undergoes significant changes, a slight compressive force (0.05 N) was applied to the material to adjust the gap height accordingly. Strain and frequency were fixed at 0.03% and 1 Hz, respectively. Under these conditions, material was assessed within its linear viscoelastic range and storage $G^{\prime }\left(T\right)$ and loss $G^{\prime \prime }\left(T\right)$ moduli or the complex viscosity, η*, could be measured.

Above 200 °C, the latter could be interpolated as a function of pulsation, ω, and temperature, T, using a classical Carreau–Yasuda model (Equation (1)):

(1) $$\left\{\begin{array}{c} \eta *\left(\omega \right)=\eta_{0}a_{T}{\left[1+{\left(\lambda a_{T}\omega \right)}^{a}\right]}^{\frac{n-1}{a}}\\ a_{T}=\exp\left[\frac{E}{R}\left(\frac{1}{T}-\frac{1}{T_{0}}\right)\right] \end{array}\right.$$

Obtained parameters were: $\eta_{0}=10,576\;Pa.s,\;n=0.3,\;a=0.46,\frac{E}{R}=4491\;K$ for $T_{0}$ of 200 °C.

To study the influence of crystallization and melting phenomena on the mechanical behavior of iPP, experimental protocols like calorimetric analyses were carried out.

Under non-isothermal conditions, the sample was initially heated up to 200 °C to erase thermomechanical history. Thereafter, the sample was subjected to successive cooling–heating cycles (200 °C → 100 °C → 200 °C) at different temperature ramp rates identical to those used in DSC (5 °C min^−1^, 3 °C min^−1^, 1 °C min^−1^ and 0.1 °C.min^−1^). Some preliminary studies showed that, for PP, the temperature difference between the rheometer thermocouple (underneath the lower plate) and the sample core is not significant up to a temperature rate of 10 °C/min, thus 5 °C/min is still relevant.

Under isothermal conditions, the sample was also heated up to 200 °C. It was then cooled down to the isothermal temperature. Due to oven thermal inertia and sample dimensions, the ramp rate cannot be as fast as in calorimetric analyses. Therefore, to enhance the control of the sample temperature and to prevent crystallization during cooling, especially when approaching the crystallization isotherm, the experimental protocol comprises two steps: cooling from 200 °C to 150 °C at 10 °C min^−1^ and then cooling from 150 °C to the crystallization isotherm at a rate of 5 °C.min^−1^. Isotherms were identical to those selected for calorimetric analyses: i.e., 125 °C, 127.5 °C, 130 °C, 132.5 °C and 135 °C. Temperature was measured under the bottom plate of the rheometer.

## 3. Theoretical

### 3.1. State of the Art

Since early works [10,11,12,13], the semi-crystalline microstructure of polymers has been described as the periodic stacks of an amorphous phase embedded between crystalline lamellae. The latter presents folding of the chains on its surface. They are connected by tie molecules. The two phases are organized in a spherical superstructure, named spherulite, formed by branched radial stacks growing from a central nucleus.

The small thickness of the crystal (order of magnitude less than 100 Å) and the chain-folding structure explain that the crystallization and melting temperatures are strongly dependent on external conditions: cooling rate and pressure. Then, three characteristic temperatures must be defined: a crystallization temperature depending on the crystallization modes, a melting temperature depending on the previous conditions and a thermodynamic equilibrium temperature $T_{m}^{0}$ supposed to be characteristic of the infinite crystal [14].

Most of the time, growth rates of radial stacks are modeled by the theory of Hoffman and Lauritzen which combines the effects of enthalpy and medium viscosity [15,16]. Despite this, and from a practical point of view, crystallization during processing is most often described at a more macroscopic level, i.e., at the level of the so-called “overall crystallization kinetics” (although the above theories are sometimes used as ingredients). Indeed, the spherical superstructure fits perfectly into this type of approach, which aims to model the fraction of volume that will be absorbed in a medium due to the nucleation and growth of uniformly distributed spheres. The Hoffman growth rate is then assimilated to the growth rate of spherulites.

Several papers have been devoted over time to overall kinetics and its uses in different conditions. A summary can be found in [17]. The most popular approach is that of Avrami [18,19,20] who corrected the work of Johnson and Mehl [21]. However, independently, the Kolmogoroff approach [22] as well as the Evans approach [23] have led to equivalent formal results.

Basically, they differ only in their mathematical treatments, but are based on the same assumptions. The theories were revised to apply them under non-uniform, non-isothermal and/or non-quiescent conditions and were validated thanks to numerical simulation (see example in general review [17]).

Applied to polymers, they allow the calculation of the evolution of the volume fraction transformed into spherulites by assuming that the spherulites are initiated on nuclei uniformly distributed and that they all grow at the same rate, which depends only on the temperature. Theories initially make no assumption on the external thermal conditions but are not easy to use in the general case. Thus, they are mainly used in their isothermal limit. Nakamura suggested that any cooling rate can be modeled using those isothermal parameters experimentally available [24,25]. Ozawa expressed Avrami’s model in the case where the cooling rate is constant [26]. This approach allows the identification of kinetics parameters in a wider range of temperatures than isothermal measurements (needed in Nakamura’s approach) and could also be used in the case of a non-constant cooling rate [27]. More recently, the differential approaches [28,29] have shown their effectiveness in the numerical modeling of the transformation but have not improved the basic assumptions.

In the present study, we used a slightly revised Evans’ approach to address crystallization kinetics. The modifications are like that of Nakamura or Ozawa. The motivation was that Evans’ way [23] of solving the problem provides some simple elements concerning spherulitic microstructure in addition to transformed volume fraction over time. This is explained in the theoretical section below. DSC allowed the estimation of Evans’ parameters and characteristics temperatures. Optical microscopy allowed the estimation of spherulite growth rate.

Regarding rheological analyses, the first step could be to correlate the moduli or viscosity to the crystallinity ratio using mixing laws [30]. Unfortunately, it is well known that crystallinity ratio alone does not allow accurate modeling of mechanical characteristics of polymers in a general manner. The inner morphology of the spherulite might be modeled and accounted for. Some attempts already exist within the frame of micromechanics that are based on simplified microstructure and that are most often restricted to an elastic behavior [31,32]. Recently, this kind of approach was coupled with numerical simulation of spherulite growth to model elastic components as a function of crystallization [33]. Nevertheless, the above developments still need some improvements and remain a scientific challenge, which justifies the use of simpler approaches while they are developed.

### 3.2. Overall Crystallization Kinetics

The theories for overall crystallization kinetics are based on the physical description we briefly recap below:

- In the molten phase, some nuclei are activated according to nucleation kinetics, i.e., a probability per unit of time that one of them will become a growing entity.
- Growth starts at once, without delay.
- Only nuclei that are in a liquid region can be activated.
- Entities stop growing when they touch, i.e., no overlapping is allowed.

The goal of the overall kinetics theories is to describe the evolution of the entities’ volume fraction (or the probability for a given point in the medium to be absorbed by one of the entities), α. It is important to note here that this quantity can be related to the degree of crystallinity if, and only if, all the spherulites possess the same constant crystallinity ratio.

In any case, the mathematical treatment would be straightforward only if the above constraints are not imposed, which would make the solution not physical. Advances in numerical solving have alleviated this difficulty, but in the 1940s some authors had to overcome this limitation in an analytical manner. So, they focused their effort on finding under what conditions and how one can link this non-physical solution to the real situation. This led to the introduction of other assumptions:

- All the entities grow at the same rate in all accessible directions (spheres in the space, disk on a plane, etc.).
- Total volume stays constant (isovolumic assumption).

Evans et al. [23] pointed out that the “non-physical problem” obeys a Poisson’s probability distribution. Then, the probability for any point to have been reached by exactly j entities at time t would be known (Equation (2)).

(2) $$p_{j}\left(t\right)=\frac{{E(t)}^{j}}{j!}exp\left(-E(t)\right)$$

E(t) is the mathematical expectancy, i.e., the average number of entities that would have been able to reach the given point between time 0 and time t. This function combines nucleation and growth kinetics.

If the second set of assumptions are followed, the probability that the point has not been reached (j = 0) is the same in both the “not physical case” and the “physical case”. This can be written as (Equation (3)):

(3) $$p_{0}\left(t\right)=\left(1-\alpha \left(t\right)\right)=exp\left(-E(t)\right)$$

E is nothing else than the extended volume fraction defined by Avrami [18,19]. So, the question is then how to quantify E. This is possible when nucleation and growth kinetics are known.

If one describes the nucleation with an activation frequency, q (probability per unit of time for one nucleus to be activated), applied to an initial density $N_{c}$ of randomly distributed pre-existing potential nuclei and giving rise to spherical entities growing at the rate $G_{c}$, one can express E and, then, $\alpha \left(t\right)$ (Equations (3) and (4)).

To achieve that point, let us consider that the probability for one nucleus per unit volume to be activated between any times τ and τ+dτ is given by $q\left(\tau \right)N\left(\tau \right)\;d\tau$, where N is the density of residual nuclei. Only those nuclei that are within less than $\left(\int_{\tau }^{t}G_{c}\left(u\right)\;du\right)$ can reach the point where E is calculated. Considering all directions in space, this defines a sphere and, finally:

(4) $$E\left(t\right)=\frac{4}{3}\pi N_{c}\int_{0}^{t}q\left(\tau \right)exp\left[-\int_{0}^{\tau }q\left(u\right)du\right]{\left[\int_{\tau }^{t}G_{c}\left(u\right)\;du\right]}^{3}d\tau$$

E is the fictitious number of entities that would have been in a situation to contribute to the crystallization of one given point representative of the medium. The density of entities that effectively exists in the medium is given in Equation (5).

(5) $$N_{a}\left(t\right)=N_{c}\int_{0}^{t}q\left(\tau \right)\left[1-\alpha \left(\tau \right)\right]d\tau$$

In the case where the product $q\times N_{c}$ is high compared to $G_{c}$ (i.e., activation is so fast that one can assume an instantaneous nucleation at the beginning of crystallization), Equations (4) and (5) can be simplified into Equation (6) and $N_{c}$ can be estimated by numbering spherulites.

(6) $$E\left(t\right)=\frac{4}{3}\pi N_{c}\int_{0}^{t}{\left[\int_{\tau }^{t}G_{c}\left(u\right)\;du\right]}^{3}d\tau$$

In such a case, only the knowledge of $G_{c}$ is needed to model crystallization. Optical microscopy or even DSC measurement [34] enables the assessment of this parameter as a function of temperature. The Hoffman–Lauritzen model [15,16] allows one to model it.

In the case $q\times N_{c}$ and $G_{c}$ have equivalent influence over time, then nucleation occurs sporadically in time, and q and $N_{c}$ have to be found. Though some attempts exist to estimate them [35], this is still difficult. At this stage, the isokinetic assumption [19,25,26] that stipulates that q and $G_{c}$ obey the same fundamental physical processes and then have the same type of dependence upon temperature (Gcq=constant) allows for the simplification of Equation (4) into Equation (7).

(7) $$E\left(t\right)=\frac{4}{3}\pi N_{c}{\left(\frac{G_{c}}{q}\right)}^{3}\int_{0}^{t}q\left(\tau \right)\;exp\left[-\int_{0}^{\tau }q\left(u\right)du\right]{\left[\int_{\tau }^{t}q\left(u\right)\;du\right]}^{3}d\tau$$

Using a characteristic time, η, defined by Avrami from q, a uniform set of equations can be proposed that is equivalent to Avrami’s set of equations (Equation (8)).

(8) $$\left\{\begin{array}{l} \eta \left(\tau \right)=\int_{0}^{\tau }q\left(u\right)du\\ E\left(t\right)=\frac{4}{3}\pi N_{c}{\left(\frac{G_{c}}{q}\right)}^{3}\int_{0}^{\eta \left(t\right)}exp\left[-\eta \left(\tau \right)\right]{\left[\eta \left(t\right)-\eta (\tau )\right]}^{3}d\eta \left(\tau \right) \end{array}\right.$$

In the case of an instantaneous nucleation, E is proportional to $\eta^{3}$ and to $\eta^{4}$ in the case of sporadic in time nucleation. In this work, we accepted the approximate form of E as given by (Equation (9)).

(9) $$E\approx k_{ok}\eta^{n}$$

If crystallization takes place in isothermal conditions or under a constant cooling rate, $\dot{T}$, and accounting for the fact that q only depends on temperature, one can easily retrieve the so-called Avrami’s or Ozawa’s equations (Equation (10)).

(10) $$\left\{\begin{array}{l} \alpha \left(t\right)\approx 1-\exp\left(-k_{Av}t^{n}\right)\\ \alpha \left(T(t)\right)\approx 1-\exp\left(-\frac{\chi \left(T\right)}{{\left|\dot{T}\right|}^{n}}\right) \end{array}\right.$$

where:

(11) $$\left\{\begin{array}{l} k_{Av}{=k_{ok}q}^{n}=k_{ok}{\left(\frac{q}{G_{c}}\right)}^{n}G_{c}^{n}\\ \chi \left(t\right)=k_{ok}{\left(\int_{T_{0}}^{T\left(t\right)}q\left(\Gamma \right)d\Gamma \right)}^{n}=k_{ok}{\left(\frac{q}{G_{c}}\right)}^{n}{\left(\int_{T_{0}}^{T\left(t\right)}G_{c}\left(\Gamma \right)d\Gamma \right)}^{n} \end{array}\right.$$

where $T_{0}$ is the initial temperature and T is the temperature at time t. $k_{Av}$ is the kinetics parameter from Avrami. n is the Avrami’s exponent. $k_{ok}$ is a kinetics parameter.

From a practical point of view, as q cannot be assessed experimentally, Equation (11) has been reformulated as a function of the growth rate. This was carried out thanks to isokinetic assumption.

### 3.3. Microstructure Description

As explained in the Introduction, some parameters that can be correlated to microstructure have been defined. The first of them could be the number of spherulites, $N_{a}$ (Equation (5)). Unfortunately, the initial density of potential nuclei, $N_{c}$, is unknown. In consequence, we decided to use N as the first parameter that is only proportional to $N_{a}$ (Equation (12)) and can be expressed as a function of α and $G_{c}$ only.

(12) $$\left\{\begin{array}{l} \begin{array}{l} N_{a}=\frac{qN_{0}}{G}\left(R-\int_{0}^{R}\alpha \left(R\right)\;dR\right)\\ N=\left(R-\int_{0}^{R}\alpha \left(R\right)\;dR\right) \end{array}\\ R=\int_{0}^{t}G_{c}\;dt \end{array}\right.$$

The second parameter is an image of the maximum average radius (Equation (13)). To calculate it, let us consider that the number of spherulites that were nucleated between times τ and τ+dτ is given by:

(13) $$dN_{a}=q\left(\tau \right)N_{c}\left(1-\alpha \left(\tau \right)\right)$$

The largest radius of those spherulites at time t is $\int_{\tau }^{t}G_{c}\left(u\right)du$. Thus, the average largest radius is:

(14) $$R_{Av}=\frac{N_{c}\int_{0}^{t}q\left(\tau \right)\left(1-\alpha (\tau )\right)\left(R(t)-R(\tau )\right)\;d\tau }{N_{a}\left(t\right)}$$

Simple mathematical treatment allowed the conclusion that $R_{Av}$ is proportional to a parameter we named <R>, that depends on $G_{c}$ and α (Equation (15)).

(15) $$<R>=\frac{\left(\frac{1}{2}R^{2}-R\int_{0}^{R}\alpha \left(R\right)\;dR+\int_{0}^{R\left(t\right)}\alpha \left(R\left(\tau \right)\right)R\left(\tau \right)\;dR\right)}{\left(R-\int_{0}^{R}\alpha \left(R\right)\;dR\right)}$$

where R is given in Equation (12).

**Figure 1:**
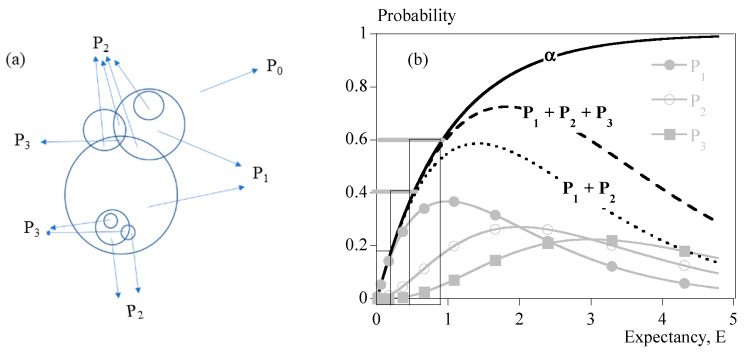
Comparison of transformed volume fraction, α, and $P_{1},\;P_{2},\;P_{3}$. (**a**) Schematic definition; (**b**) probability vs. mathematical expectancy, E.

The latter parameters are deduced from Evans’ approach. Indeed, it is possible to know the probability for one point to be absorbed by exactly two entities in the fictitious case of a Poisson’s probability distribution, $P_{2}$ (Equation (2)). One can assume that as long as $P_{2}$ is negligible, transformation mainly results from $N_{a}$ individual spherulites of maximum radius R. The transformed volume fraction is almost equal to $P_{1}$. When $P_{2}$ becomes significant, $\alpha \approx P_{1}+P_{2}$. At this stage, the presence of aggregates of two spherulites becomes probable, although it is not possible to give an exact number. The same demonstration can be carried out with $P_{3}$ that enables one to know whether aggregates of three spherulites are probable or not. Figure 1 depicts the meaning of those parameters.

To summarize:

- Below 10 to 15% transformed volume fraction (α), crystallization results mainly from the growth of statistically individual spherulites. During that period, the microstructure can be seen as $N_{a}$ individual semi-crystalline spheres per unit volume in a soft matrix. The largest radius is R. The size distribution and the average radius depend on nucleation kinetics.
- From 15 to 40%, impingement of spherulites cannot be neglected. Obviously, Poisson’s analysis does not allow evaluation of the number of impingements. It only allows one to say that it is probable that impingements had occurred. Then, microstructure results from single spherulites and isolated aggregates.
- From 40%, presumably, coalescence takes place. Progressively, the microstructure turns into a solid “skeleton” with embedded liquid pockets.

Figure 1 depicts the result of this analysis as a function of mathematical expectancy, E, making it a universal analysis that does not depend on nucleation and growth parameters. We used this picture to estimate the topology of the microstructure at the instant of a significant increase in the viscosity.

## 4. Results and Discussion

### 4.1. Methodology

Isothermal measurements were used to draw a hypothesis and propose a pathway for models that were tested with experiments under constant cooling rates. We provide a brief summary of the approach that was then developed:

- First, $G_{c}$ was measured thanks to optical microscopy as a function of the temperature. It was modeled with the Hoffman and Lauritzen equation [36,37] (Equation (16)):

(16) $$G_{c}=G_{0C}exp\left(-\frac{U^{*}}{8.32\left(T-T_{\infty }\right)}\right)exp\left(-\frac{K_{g}}{T\left(T_{m}^{0}-T\right)}\right)$$

U* was taken at 6270 J/mol and $T_{\infty }$ at −21 °C according to the literature [36]. $T_{m}^{0}$ was 208 °C and $\Delta H_{\infty }$ was 148 J.g^−1^ [38]. $G_{0c}$ and $K_{g}$ were estimated from our measurements to be 4.5 × 10^8^ µm/min and 6.34 × 10^5^ K^2^, respectively. The value for $K_{g}$ was found to be in rather good agreement with the literature (7.28 × 10^5^ K^2^ to 8.66 × 10^5^ K^2^ after [38]).

- Second, the isothermal crystallizations were performed using DSC and the transformed volume fraction, α, was recorded as a function of time and temperature.
- Third, the parameters, N (Equation (12)) and <R> (Equation (15)) were calculated thanks to numerical integration. For their parts, $P_{1}$ and $P_{2}$ were deduced from α.
- Fourth, isothermal rheological measurements were correlated to the parameters (see section below). A model was deduced. At this stage, storage and loss moduli were considered independently.
- Fifth, Avrami’s kinetics parameter and exponent (i.e., $k_{Av}$ and n) were deduced from α. n was averaged at 3 and $k_{ok}{\left(\frac{q}{G}\right)}^{n}$ was estimated from $G_{c}$ and α (Equations (10) and (11)). Results are presented in Table 1.
- Finally, $k_{ok}{\left(\frac{q}{G}\right)}^{n}$ was averaged at 1.96 × 10^−6^ µm^−3^ min^3^ to model crystallization under a constant cooling rate. Both the rheology and crystallization models were merged to assess extrapolation of results to predict rheological measurements carried out under constant cooling and heating rates.

In parallel, the Ozawa’s model (Equation (10)) was applied to tests performed under constant cooling rates, and the function χ was estimated for a value of n equal to 3 (Equation (17), following [27].

(17) $$ln\left(\chi \right)=96.112-0.7676\;T({}^{\circ}C)$$

**Figure A4:**
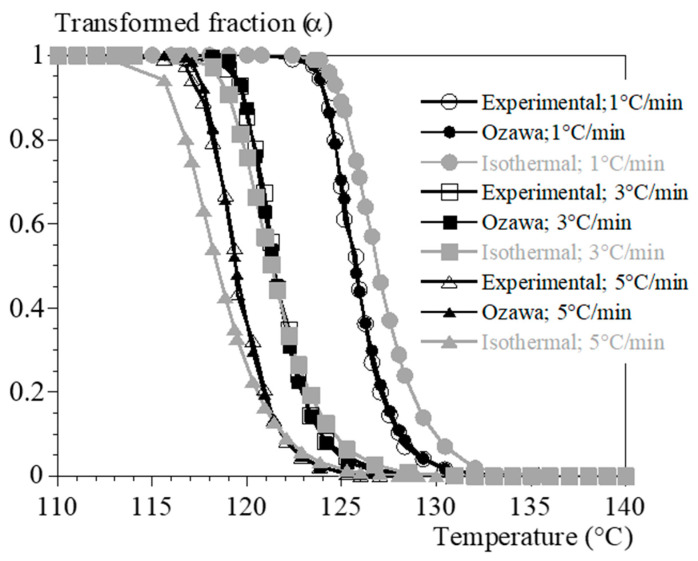
Crystallization kinetics under constant cooling rates. Comparison between experimental data (hollow black symbols), identification with Ozawa’s model [26] (filled black symbols) and identification from isothermal data (Table 1, filled grey symbols).

The respective effectiveness of the two identifications can be compared in Figure A4 in Appendix A. In the following, the first route is referred to as the “Avrami-like” approach while the second is referred to as Ozawa’s model. To conclude, the first one (from isothermal measurements) should allow the estimation of orders of magnitude while Ozawa’s model is accurate. In our context, these estimations will be used as input data to validate rheological models in realistic conditions (i.e., in conditions where crystallization kinetics cannot be measured in situ).

### 4.2. Rheology in Isothermal Conditions

**Figure 2:**
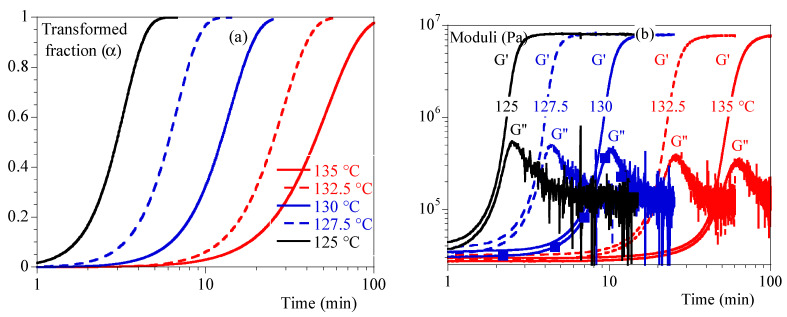
Isothermal crystallization: comparison between 125, 127.5, 130, 132.5 and 135 °C. (**a**) Transformed volume fraction vs. time. (**b**) Storage modulus, G′, and loss modulus, G″, vs. time.

Isothermal evolutions of the transformed volume fraction, and of the storage G′ and loss G″ moduli, are illustrated in Figure 2a,b.

A mechanical stiffening was seen contemporaneously with crystallization observed in DSC. However, the events did not appear to be simultaneous in all cases. In fact, it appeared that the mechanical response was postponed compared to the thermal ones. As no artifacts could be found, it could be concluded that mechanical measurements were sensitive to crystallization but not totally driven by the transformed volume fraction or crystallinity ratio as they are related.

**Figure 3:**
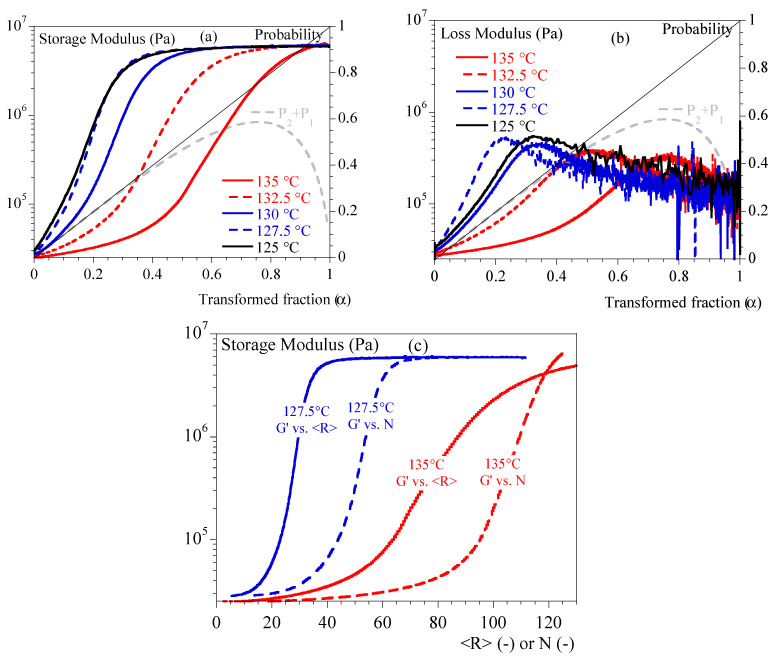
Isothermal crystallization: comparison between 125, 127.5, 130, 132.5 and 135 °C. (**a**) Storage modulus vs. transformed fraction. (**b**) Loss modulus vs. transformed fraction. Straight line represents transformed fraction. Dashed grey line is $P_{1}+P_{2}$ (Equation (2)). (**c**) Storage modulus vs. <R> (solid lines) and N (dashed lines) for two temperatures (127.5 and 135 °C).

Figure 3 depicts G′ and G″ as a function of α. Depending on temperature, we can notice that mechanical stiffening took place between transformed fractions of 10% and 45%. This difference was correlated to the temperature: the lower the temperature, the more rapid the stiffening. G″ showed a peak followed by a relaxation over time. The maximum peak appeared for transformed fraction of 20% at 127.5 °C and 80% at 135 °C. In conclusion, the transformed volume fraction is enough to describe the mechanical state of the material.

In Figure 3, the evolution of Poisson probability as a function of α is plotted. We can notice that crystallization at a low temperature is such that mechanical evolution could be seen during the first stage of crystallization: single spherulites and isolated small aggregates. Conversely, crystallization at a higher temperature implies a material close to coalescence of spherulites.

As a first approach, storage modulus versus transformed fraction data have been described using the general effective medium (GEM) law. This analysis aimed to find a parameter equivalent to a mechanical percolation threshold, specifically the transformed fraction at which a percolating cluster of spherulites appears. This law allows fitting of both parts of the curve (below and above the percolating threshold) at the same time. It was initially used to describe the electrical percolation phenomenon [39] but has been recently adapted to describe percolation of spherulites during crystallization processes [40]. The GEM equation is as follows (Equation (18)):

(18) $$\left\{\begin{array}{c} \begin{array}{l} \left(1-\alpha \right)\frac{{G_{0}^{\prime }}^{1/s}-{G^{\prime }}^{1/s}}{{G_{0}^{\prime }}^{1/s}+A.{G^{\prime }}^{1/s}}+\alpha \frac{{G_{\infty }^{\prime }}^{1/t}-{G^{\prime }}^{1/t}}{{G_{\infty }^{\prime }}^{1/t}+A.{G^{\prime }}^{1/t}}=0\\ A=\frac{1-\alpha_{c}}{\alpha_{c}} \end{array} \end{array}\right.$$

$\alpha_{c}$ is the percolation threshold in terms of transformed fraction. $G_{0}^{\prime }$ is the storage modulus in the melting state (i.e., at α=0). $G_{\infty }^{\prime }$ is the storage modulus in solid state (i.e., at α=1). Exponent s describes the slope of the curve below the percolation threshold, while exponent t describes the slope of the curve above the percolation threshold. Using this equation as it stands resulted in satisfactory agreements with experimental data. However, the fit parameters obtained were not physically consistent: e.g., the percolation threshold value at 125 °C was estimated at a transformed fraction of 1.1. Therefore, it seemed that this law was not totally adapted to the studied system and, therefore, it was chosen to investigate another phenomenological approach.

Before moving forward, let us notice that, in Figure 3, it is also visible that the measurement at 125 °C appeared to be different from the others, whereas it did not reveal any specificity when analyzed as a function of time (Figure 2). Indeed, it is well known that isothermal crystallization at low temperatures is always tricky as crystallization can begin during the cooling step prior to the test. In our case, crystallization began at a higher temperature than 125 °C, during that cooling phase that was not recorded. Let us emphasize that mechanical curves are shifted towards higher temperatures which is a kind of validation.

**Figure 4:**
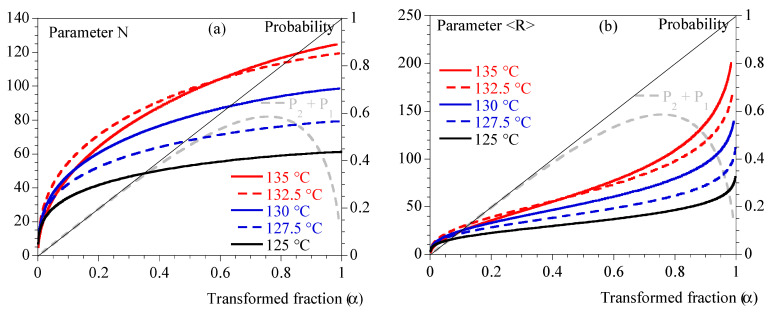
Isothermal crystallization: comparison between 125, 127.5, 130, 132.5 and 135 °C. (**a**) N (Equation (12)) vs. transformed fraction. (**b**) <R> (Equation (15)) vs. transformed fraction. Straight line represents transformed fraction. Dashed grey line is $P_{1}+P_{2}$ (Equation (2)).

Finally, Figure 4 depicts N and <R> and illustrates that the different conditions of crystallization did not involve the same number of spherulites or the same maximum average radius.

### 4.3. Rheology under Non-Isothermal Conditions

**Figure 5:**
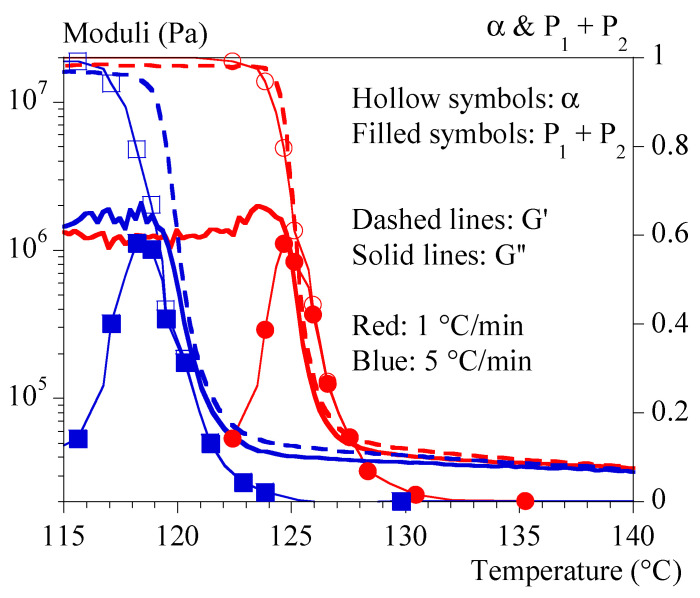
Mechanical properties during crystallization performed under constant cooling rates. Comparison between 1 (blue) and 5 °C/min (red). Storage modulus, G′, is represented with dashed lines, loss modulus, G″, with solid lines. Symbols represent α (hollow symbols) and $P_{1}+P_{2}$ (filled symbols).

**Figure 6:**
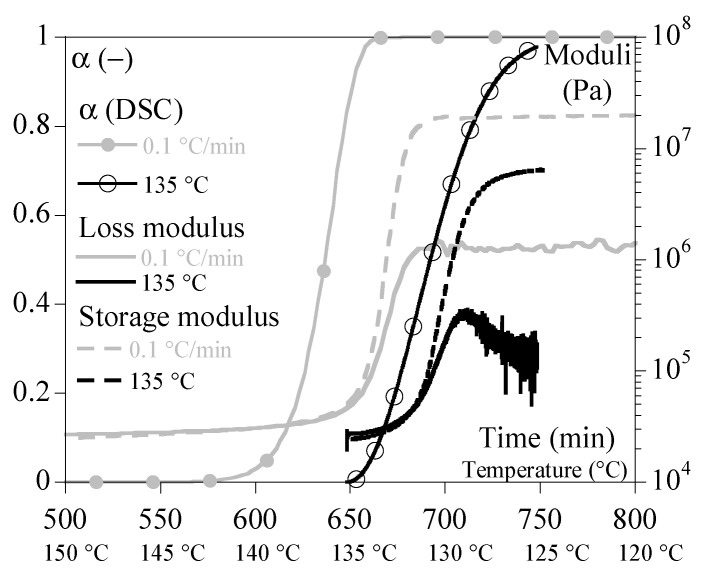
Mechanical properties during crystallization performed under constant cooling rates at 0.1 °C/min (grey) compared to isothermal crystallization at 135 °C (black). Storage modulus, G′, is represented with dashed lines, loss modulus, G″, with solid lines. Symbols stand for α.

Figure 5 and Figure 6 show typical results obtained under non-isothermal conditions (i.e., at constant cooling rates of 0.1 and 5 °C/min, respectively). Figure 5 groups the tests performed at 1 and 5 °C/min by superimposing the evolutions of G′, G″ and α. It allows drawing of the same general conclusions as those obtained under isothermal conditions.

In summary, the mechanical effects of crystallization are contemporaneous with the thermal effects but not completely simultaneous. Stiffness may involve different stages of crystallization depending on the thermal conditions. The higher the crystallization temperature, the more the last stages are involved (high $P_{1}+P_{2}$). Schematically, the crystallizing medium can appear as composed of individual spherulites whose number and radius vary for low crystallization temperatures (high cooling rates) or coalescing spherulites for higher temperatures (low cooling rates). Based on what is known in the field of micromechanics [32,33], this should have a significant impact and make a simple correlation with the crystallinity rate irrelevant. This may also explain the apparent inconsistency of results in the literature regarding this correlation.

Figure 6 superimposes the results of the tests performed under a cooling rate of 0.1 °C/min, which led to a crystallization temperature of 135 °C and the one performed at constant temperature of 135 °C. To compare isothermal and non-isothermal results, the time scale of the isothermal test has been shifted for the drawing. This superposition shows that there is a certain level of similarity between the two conditions but that the cooling phase before crystallization is important. In other words, the thermal history before crystallization plays a role. In addition, the isothermal conditions allow observation of some relaxation, mainly of G″, after crystallization which is either hidden or non-existent during controlled cooling.

We can try to reformulate these conclusions as follows. The thermodynamics and kinetics of nucleation and growth define the temperature at which the level of crystallinity increases. This temperature controls the contrast in behavior between the two phases. We cannot exclude the possibility that the first stages of crystallization lead to an imperfect ordered phase whose intrinsic properties would evolve with time. At this stage, we do not have any information going in this direction or that are contradictory.

The fact remains that an attempt at modeling must integrate parameters characteristic of the microstructure and of the contrast of properties. This is why the four parameters N, <R>, α and $P_{1}+P_{2}$ have been defined above.

## 5. Modeling

### 5.1. Methodology

The goal of this modeling part is the development of a phenomenological interpolation for G′ and G″ based on the chosen characteristic parameters. The two moduli were considered independently. Isothermal measurements were first considered to suggest formalisms.

Then, parameters were identified with the solver of EXCEL^®^ software with a mean square cost function. Identification was possible with all the tests taken one by one. Parameters from isothermal tests showed coherent evolution with temperature, except the one at 125 °C that crystallized during the cooling step. To better address dependence upon temperature, it was, then, useful to add one non-isothermal test to the database. We chose a test performed at a low cooling rate (0.1 °C/min) to stay close to isothermal conditions.

Other experiments under constant cooling rates were used as validation trials. In that step, the transformed volume fraction was also numerically estimated from our identified kinetics. The two estimates (from Avrami’s and from Ozawa’s equations) were considered.

### 5.2. Rheology vs. Crystallization

Basic assumptions were that G′ should be controlled by the microstructure and the temperature, while G″, that is related to dissipation, should be controlled by the amount of amorphous phase and by the mobility of the chains inside it. To account for the latter assumption, some parameters were assumed to depend on temperature in the same manner as the growth rate, imagining that the same fundamental motions should be involved. This was carried out by mimicry with an isokinetic assumption. For convenience, we assumed we can write those parameters as a function of the growth rate of spherulites, $G_{C}$. Best fits were obtained with formalisms given in Equation (19).

(19) $$\left\{\begin{array}{l} G^{\prime }=G_{L}^{\prime }+0.5\left(G_{S}^{\prime }-G_{L}^{\prime }\right)\left(1+\mathrm{Tanh}\left(C_{R}^{\prime }{<R>}^{n_{R}}+C_{E}^{\prime }\left(P_{1}+P_{2}\right)-C_{N}^{\prime }N-C_{T}^{\prime }\right)\right)\\ G^{\prime \prime }=G_{L}^{\prime \prime }\left(1-\alpha \right)+\left(G_{c}^{\prime \prime }X_{c}+G_{a}^{\prime \prime }\left(1-X_{c}\right)\right)\alpha +C_{E1}^{\prime \prime }\frac{E^{n_{1}}}{1+{\left(C_{E2}^{\prime \prime }E\right)}^{n_{2}}} \end{array}\right.$$

$G_{L}^{\prime }$ in Equation (19) corresponds to the storage modulus of PP when the crystallinity ratio is zero. $G_{S}^{\prime }$ is the storage modulus when solid (spherulites). Equation (19) expresses the fact that the storage modulus, G′, gradually increases from $G_{L}^{\prime }$ to $G_{S}^{\prime }$ when crystallization occurs in a manner that should depend on the number and the radius of the spherulites, the level of impingements between them as well as the temperature (if parameters depend on temperature).

The purpose of Equation (19) is to fit experimental data, so its shape results from mathematical considerations. Figure 3a,c illustrates that, at a given temperature, G′ varies with transformed fraction, <R> and N in similar manners, i.e., through sigmoidal-like curves. Dependence upon temperature results in a shift in the inflection points of the “sigmoids” and a change in the slope of these latter. For convenience, this general shape was reproduced thanks to the general form (1 + Tanh(X)) which is equal to 0 for low values of X (liquid) and equal to 2 for higher values of X (solid). So, X must be a function that increases during crystallization, depends on our chosen parameters and reproduces the shift in inflection point. We first tested the simplest solution, which is when X depends on <R>, $\left(P_{1}+P_{2}\right)$ and N according to a multilinear function, which minimized the number of parameters. This allows the definition of three main parameters to be adjusted by temperature: $C_{R}^{\prime },C_{E}^{\prime }\;\mathrm{and}\;C_{N}^{\prime }$. Power $n_{R}$ appeared to be useful to improve the fit during the trials. In addition, $C_{T}^{\prime }$ of Equation (19) contributes ruling the inflection point whose location depends on T (when $C_{R}^{\prime }{<R>}^{n_{R}}+C_{E}^{\prime }\left(P_{1}+P_{2}\right)-C_{N}^{\prime }N=C_{T}^{\prime }$).

Alternatively, $G_{L}^{\prime \prime }$ is the loss modulus of the liquid. That of the solid PP, $G_{S}^{\prime \prime }$, should depend on the crystallinity ratio (0.54 in this paper), $X_{c}$, the loss modulus of the crystal, $G_{c}^{\prime \prime }$, and the loss modulus of the amorphous phase included in the spherulites, $G_{a}^{\prime \prime }$. Equation (19) expresses that G″ results from the combination of dissipation in the crystal, that in the liquid and that in the amorphous phase, basically according to mixing laws with some disturbance effects due to microstructure (term in $C_{E1}^{\prime \prime }\frac{E^{n_{1}}}{1+{\left(C_{E2}^{\prime \prime }E\right)}^{n_{2}}})$. The latter are expressed as a function of Evans expectancy to mimic $P_{j}$.

$G_{L}^{\prime }$ and $G_{L}^{\prime \prime }$ were estimated from rheological measurements and were not identified with other parameters in our calculations. Indeed, the experimental sets were too limited to allow their identification from isothermal trials.

Parameters to identify are, then: $G_{S}^{\prime }{,\;C}_{R}^{\prime },\;n_{R},\;C_{E}^{\prime }{,\;C}_{N}^{\prime }{,\;C}_{T}^{\prime }$ on one hand (G′) and $G_{c}^{\prime \prime }{,\;G}_{a}^{\prime \prime },\;C_{E1}^{\prime \prime },\;n_{1},\;C_{E2}^{\prime \prime }$ and $n_{2}$ on the other hand (G″). Data resulting from identification from tests, one by one and combined, are gathered in Table 2.

### 5.3. Rheology vs. Melting

Melting results from a progressive disappearance of lamellae, keeping the spherulitic superstructure unchanged. So, it was considered that storage and loss moduli during melting results from a mixing law (Equation (20)).

(20) $$\left\{\begin{array}{l} G^{\prime }=G_{L}^{\prime }\left(1-X_{c}\alpha \right)+G_{c}^{\prime }X_{c}\alpha \\ G^{\prime \prime }=G_{L}^{\prime \prime }\left(1-X_{c}\alpha \right)+G_{c}^{\prime \prime }X_{c}\alpha \end{array}\right.$$

For simplicity in this paper, we only used data for heating rates of 1 °C/min, i.e., Arrhenius-like functions:

(21) $$\left\{\begin{array}{l} G_{L}^{\prime }\left(Pa\right)=10^{5};\;G_{c}^{\prime }\left(Pa\right)=10^{7}\exp\left(\frac{500}{T(K)}\right)\\ G_{L}^{\prime \prime }\left(\mathrm{Pa}\right)=227\;\exp\left(\frac{2042}{T(K)}\right);\;G_{c}^{\prime \prime }\left(\mathrm{Pa}\right)=2430\;\exp\left(\frac{2667}{T(K)}\right) \end{array}\right.$$

## 6. Results

**Figure 7:**
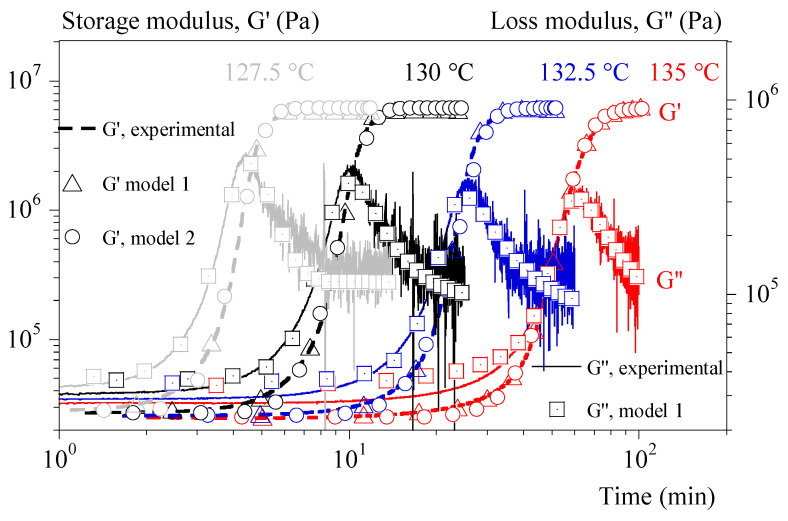
Storage and loss moduli (G′ and G″) vs. time during isothermal crystallization. Comparison between experiments (dashed and solid lines) and calculus. Calculus 1 is based on identification one by one. Calculus 2 combines isothermal tests and tests under 0.1 °C/min.

Equation (19) and the parameters of Table 2 make possible to reproduce the isothermal evolution of G′ and G″ well whatever the protocol (Figure 7). Nevertheless, only identification at 0.1 °C/min could be extrapolated to non-isothermal tests.

**Figure 8:**
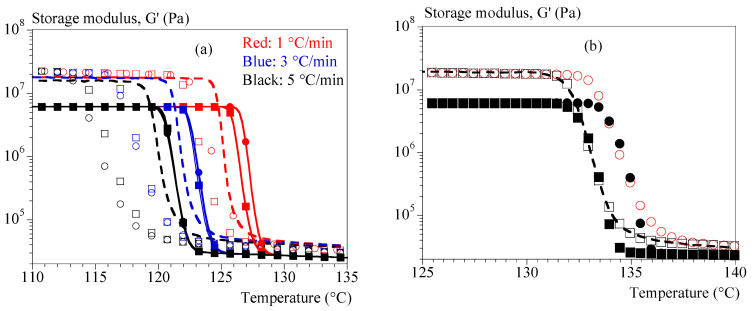
Validation of Equation (18) for storage modulus, G′. (**a**) Comparison between experiments (dashed lines) and calculus (symbols). Colors refer to cooling rates; hollow squares: identification with tests at 0.1 °C/min and Ozawa crystallization; hollow circles: identification with tests at 0.1 °C/min and Avrami-like crystallization; filled squares: combined with isothermal test identification and Ozawa crystallization; filled circles: combined with isothermal test identification and Avrami-like crystallization. (**b**) Results of a cooling rate of 0.1 °C/min.

Figure 8a displays the experimental (dashed lines) and calculated (symbols) evolutions of modulus G′ as a function of temperature for cooling rates of 1, 3 and 5 °C/min. These tests were not used when finding the parameters, this is therefore a validation. Only the formalism of Equation (19) was used. Parameters were found in two ways:

1- by using only the test at 0.1 °C/min and interpolating the crystallization kinetics with Ozawa’s formalism (Equation (10));
2- by combining the test at 0.1 °C/min and the isothermal tests and interpolating the kinetics of not isothermal crystallization with the formalism of Ozawa (Equation (10)).

Validations were adapted in four ways:

- by using the coefficient 1 under the conditions of identification 1 (Ozawa crystallization, hollow squares);
- by using the coefficient 1 and interpolating the crystallizations during the validation tests from the isothermal tests (hollow circles);
- by using the coefficient 2 under the conditions of identification 2 (Ozawa crystallization, filled squares);
- by using the coefficient 2 and interpolating the crystallizations during the validation tests from the isothermal tests (filled circles).

Figure 8b depicts the efficiency of those four routes for the test under a cooling rate of 0.1 °C/min.

Our approach reproduces the effect of the cooling rate and the order of magnitude of the moduli.

**Figure A5:**
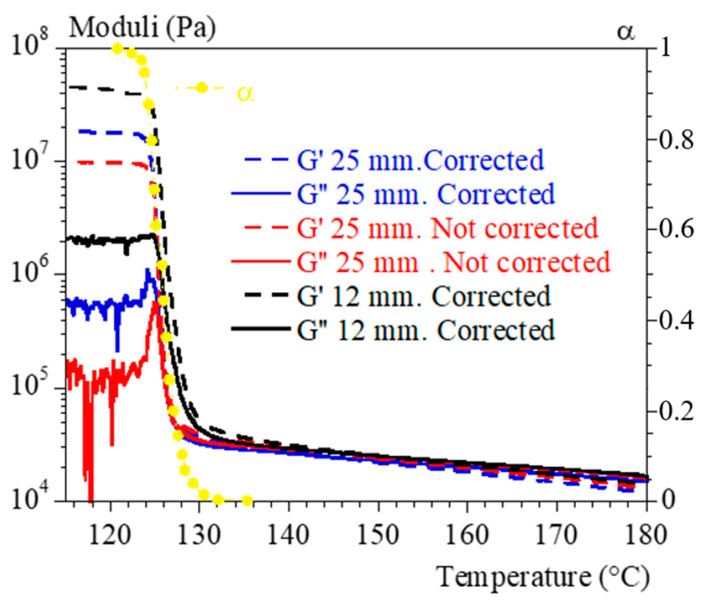
Evolution of G′ and G″ vs. temperature for a cooling rate of 1 °C/min. Comparison between two geometries (25 and 12 mm) and assessment of manufacturer compliance correction. Yellow line represents evolution of transformed volume fraction for the 25 mm geometry.

However, predictions of the rate effect are clearly better if the identification is carried out on a larger data set (identification 2). In parallel, it seems that a better estimation of the crystallization kinetics is favorable (Ozawa model). Finally, the model would need to be adjusted to better estimate the modulus after crystallization. However, to achieve that point it will be necessary to consider the relaxations specific to the amorphous phase. In addition, it is recognized that tests can need to be corrected to consider the lack of rigidity of rheometers, especially at high torques, i.e., when the material is solid [41,42,43]. To assess the need for correction in our case, we compared the results of a test (1 °C/min) with and without the manufacturer’s correction with a test using a smaller geometry (Figure A5). It could be concluded that the instrument is not rigid enough to accurately characterize solid polymers without a correction. The correction provided by the manufacturer does not seem to be sufficient. However, only the last part of the test is significantly affected (above 70% transformed volume fraction). This means that the shape of the curves is not sensitive to correction. The possible low stiffness of the device does not therefore call into question the discussions or the shape of the proposed laws. Only the solid plateau value should be sensitive to this artifact. As was said, this parameter needs to be improved to account for solid transitions in the approach. The transition temperature seems to be shifted by 2 °C with the small plateau, but we also know that the cooling kinetics is stronger in this case. When changing the measurement geometry, we should have recalibrated the thermal kinetics. At this stage, the approach is effective, but the identification of solid-specific parameters and their temperature evolution requires further work, the first stage of which will be a rigidity analysis of the device. Further study is therefore still needed.

**Figure 9:**
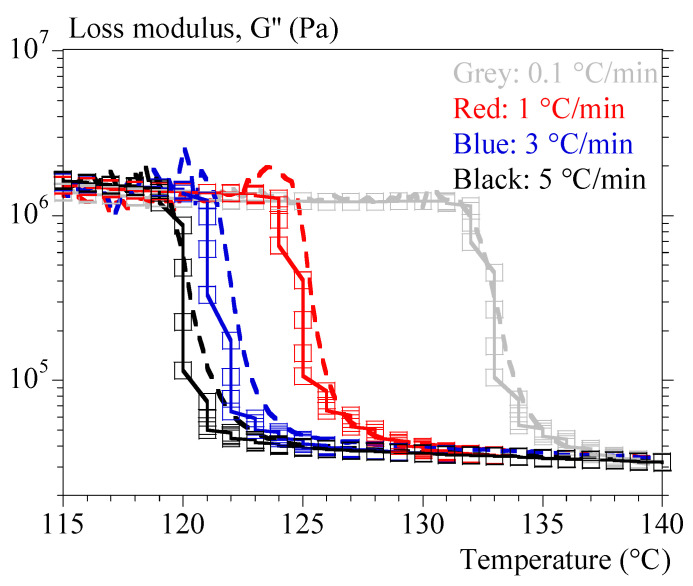
Validation of Equation (18) for loss modulus, G″. Comparison between experiments (dashed lines) and calculus (symbols). Colors refer to cooling rates; hollow squares: identification with tests at 0.1 °C/min and Ozawa crystallization.

Regarding G″, the proposed formalism was able to independently reproduce the isothermal and the non-isothermal tests, but a unique set of parameters was not found. This difficulty in reproducing the two conditions through a single set of parameters is not surprising. G″ contains the viscoelastic effects and has therefore a particular relation to time. We could, however, propose a modeling of the cooling rate effect that was quite satisfactory from identification with the test at 0.1 °C/min (Figure 9).

**Figure 10:**
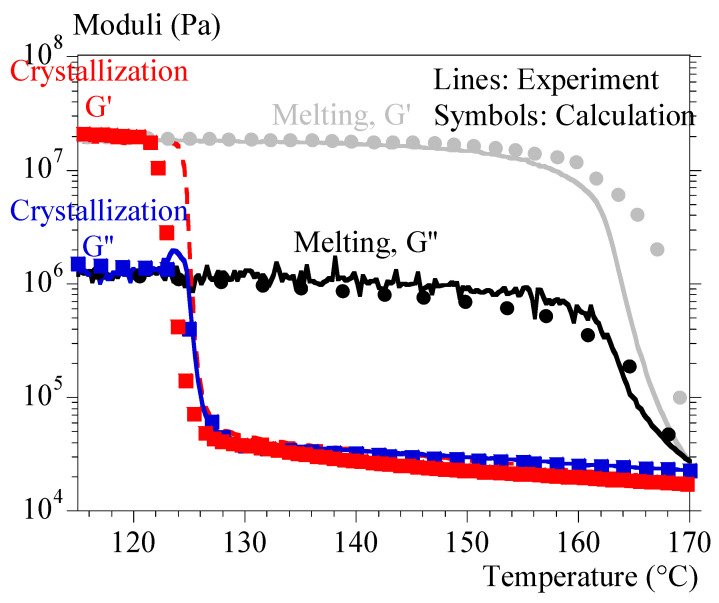
Heating–cooling cycle at 1 °C/min; experiments–calculation comparison.

As for the melting tests, they were sufficiently well reproduced with the simple mixing law. It was then possible to reproduce melting–crystallization cycles with a good agreement (Figure 10).

Note, however, that each condition was treated independently, assuming that the initial states were at equilibrium: total and relaxed melting and complete crystallization at equilibrium. Cycle modeling will require considering intermediate crystalline states. In the same way, a complete modeling now requires accounting for the relaxations specific to the amorphous phase, in particular the principal relaxation. This will require further work.

Let us conclude here that the feasibility of the approach is confirmed. It is a pragmatical and simple alternative to the necessary theoretical developments.

## 7. Conclusions

The linear properties of PP during crystallization were studied under isothermal conditions and at constant cooling rates with the aim of helping the modeling of viscoelasticity during crystallization and melting of polymers. PP was chosen as model material. The results and discussions allow us to suggest some routes concerning the parameters to be accounted for in the future and to develop a phenomenological approach to estimate the correlation between crystallization and linear viscoelasticity.

It was shown that, whatever the conditions, the mechanical effects of crystallization cannot be modeled by considering the degree of crystallinity or the transformed fraction alone. This is a clear explanation of the controversial results in the literature that relied on this assumption.

In fact, the mechanical effects of crystallization are contemporaneous with the increase in the transformed volume fraction but not simultaneously in all cases. Stiffening can occur at various stages of crystallization, depending on the thermal conditions. This implies that behavior is not a result of the number or size of spherulites only.

In summary, the higher the crystallization temperature, the more coalescence between spherulites is involved when the modulus increases.

Consequently, the crystallizing medium must be considered for individual spherulites at low temperature (high cooling rate), aggregates at medium temperature or coalescing spherulites at elevated temperature (low cooling rate) for the storage and loss moduli to change significantly. This is a real improvement in knowledge in the field of micromechanics.

One reason for this could be that the contrast in the behavior between the two phases is of paramount importance in characterizing the properties of the crystallizing medium.

This justifies more in-depth studies aiming at combining microstructure modeling and micromechanical modeling of the mechanical behavior of a heterogeneous medium.

In a first approach, we show that one can refer to the microstructure at the spherulite scale in a simplified way to suggest phenomenological models for the storage and loss moduli.

This represents a second important result of the study. It has been possible to propose simple mathematical models of the evolution of the two moduli as a function of the temperature for different cooling rates, which requires only classical crystallization analyses, growth rate measurements and rheometric tests to be used.

This could help to estimate residual stresses in additive manufacturing until more rigorous models are developed.

## Figures and Tables

**Table 1:** Isothermal characteristics for crystallization. Parameters are defined in Equations (10) and (11).

Temperature (°C)	125	127.5	130	132.5	135
$G_{c}$ (µm/min)	20.8	13.2	7.93	4.68	2.69
$k_{Av}$ (min^−n^)	1.77 × 10^−2^	2.46 × 10^−3^	7.08 × 10^−4^	1.59 × 10^−4^	2.52×10^−4^
N	3.39	3.15	2.75	2.60	2.09
$k_{Av}$ for n = 3 (min^−3^)	4.00 × 10^−5^	8.33 × 10^−5^	9.12 × 10^−4^	4.51 × 10^−3^	2.81 × 10^−2^
$k_{ok}{\left(\frac{q}{G_{c}}\right)}^{n}$ (µm^−3^ min^3^)	3.13 × 10^−6^	2.00 × 10^−6^	1.78 × 10^−6^	7.96 × 10^−7^	2.10 × 10^−6^

**Table 2:** Parameters of Equation (18) to express G′ and G″ as a function of T.

		135 (°C)	132.5 (°C)	130 (°C)	127.5 (°C)	125 (°C)	0.1 °C/min	Combined
	
$G_{L}^{\prime }$ (MPa)		$40.43\times 10^{-6}\;\exp\left(\frac{2623.3}{T(K)}\right)$						
$G_{S}^{\prime }$ (MPa)		7.04	6.16	5.98	5.86	5.98	0.22 $\exp\left(\frac{1776.4}{T(K)}\right)$	6.64 $\exp\left(\frac{-29.8}{T(K)}\right)$
$C_{R}^{\prime }\left({\mu m}^{-n_{R}}\right)$		3.99	3.14	2.01	1.85	1.93	0.23	0.767 $\exp\left(\frac{2352}{T(K)}\right)$
$n_{R}$ (-)		0.323	0.355	0.408	0.447	0.455	0.71	0.023 $\exp\left(\frac{-31.6}{T(K)}\right)$
$C_{R}^{\prime }$ (-)		0.0879	0.0663	0.0593	0.059	0.0544	−0.035	−276 $\exp\left(\frac{-3310}{T(K)}\right)$
$C_{T}^{\prime }$ (-)		11.4	10.6	8.22	8.05	8.04	4.28	1.35 $\exp\left(\frac{-2154}{T(K)}\right)$
$C_{E}^{\prime }$ (-)		4.18	4.67	6.96	8.16	6.03	0.24	0.0015 $\exp\left(\frac{2971}{T(K)}\right)$
$G_{L}^{\prime \prime }$ (MPa)		$2.27\times 10^{-4}\;\exp\left(\frac{2042}{T(K)}\right)$						
$G_{c}^{\prime \prime }$ (MPa)		0.0936	0.1082	0.1254	0.1800	0.0809	5.32 × 10^−12^ $\exp\left(\frac{9625}{T(K)}\right)$	NA
$G_{a}^{\prime \prime }$ (MPa)		0.047	0.048	0.050	0.051	0.053	2.28 × 10^−4^ $\exp\left(\frac{-2171}{T(K)}\right)$	NA
$C_{E1}^{\prime \prime }$ (-)		199,577	3,943,412	18,398,585	20,373,492	16,690,372	6.57 $\exp\left(0.335\;G_{c}\right)$	NA
$n_{1}$ (-)		5.03	3.77	3.21	2.44	2.87	3.97	NA
$C_{E2}^{\prime \prime }$ (-)		0.848	1.752	2.897	3.692	2.977	0.0485 $\exp\left(0.083\left(G_{c}-0.194\right)\right)$	NA
$n_{2}$ (-)		6.95	5.22	4.73	4.54	3.91	3.99	NA

## Data Availability

Not applicable.

## References

[1] Biskas H., Stavropoulos P., Chryssolouros G. (2016). Additive manufacturing methods and modelling; a critical review. Int. J. Adv. Manuf. Technol..

[2] Haudin J.-M., Boyer S.A.E. (2017). Crystallization of Polymers in Processing Conditions: An Overview. Int. Polym. Process..

[3] Sreejith P., Kannan K., Rajagopal K.R. (2023). A thermodynamic framework for the additive manufacturing of crystallizing polymers. Part I: A theory that accounts for phase change, shrinkage, warpage and residual stress. Int. J. Eng. Sci..

[4] Boutahar K., Carrot C., Guillet J. (1998). Crystallization of polyolefins from rheological measurements–relation between the transformed fraction and the dynamic moduli. Macromolecules.

[5] Pogodina N.V., Winter H.H. (1998). Crystallization as a Physical Gelation Process. Macromolecules.

[6] Lamberti G., Peters G., Titomanlio G. (2007). Crystallinity and Linear Rheological Properties of Polymers. Int. Polym. Proc..

[7] Han S., Wang K.K. (1997). Shrinkage prediction for slowly-crystallizing thermoplastic polymers in injection molding. Int. Polym. Proc..

[8] Pantani R., Speranza V., Titomanlio G. (2015). Simultaneous morphological and rheological measurements on polypropylene: Effect of crystallinity on viscoelastic parameters. J. Rheol..

[9] Aris-Brosou M., Vincent M., Agassant J.-F., Billon N. (2017). Viscoelastic rheology in the melting and crystallization domain: Application to polypropylene copolymers. J. Appl. Polym. Sci..

[10] Keith H.D., Vadimsky R.G., Padden F.J. (1970). Crystallization of isotactic polystyrene from solution. J. Polym. Sci. Part. A-2 Polym. Phys..

[11] Keith H.D., Padden F.J., Vadimsky R.G. (1971). Intercrystalline links: Critical evaluation. J. Appl. Phys..

[12] Padden F.J., Keith H.D. (1973). Mechanism for lamellar branching in isotactic polypropylene. J. Appl. Phys..

[13] Keith H.D., Padden F.J. (1984). Twisting orientation and the role of transient states in polymer crystallization. Polymer.

[14] Hoffman J.D., Weeks J.J. (1962). Melting process and the equilibrium melting temperature of polychlorotriuoroethylene. J. Res. Nat. Bur. Stand. Sect. A Phys. Chem..

[15] Hoffman J.D., Miller R.L. (1997). Kinetics of crystallization from the melt and chain folding in polyethylene fractions revisited: Theory and experiment. Polymer.

[16] Lauritzen J.I., Hoffman J.D. (1973). Extension of theory of growth of chain-folded polymer crystals to large undercoolings. J. Appl. Phys..

[17] Piorkowskaa E., Galeski A., Haudin J.-M. (2006). Critical assessment of overall crystallization kinetics theories and predictions. Prog. Polym. Sci..

[18] Avrami M. (1939). Kinetics of phase change. I. General theory. J. Chem. Phys..

[19] Avrami M. (1940). Kinetics of phase change. II. Transformation–time relations for random distribution of nuclei. J. Chem. Phys..

[20] Avrami M. (1941). Kinetics of phase change. III. Granulation, phase change and microstructure. J. Chem. Phys..

[21] Johnson W.A., Mehl R.F. (1939). Reaction kinetics in process of nucleation and growth. Trans. AIME.

[22] Kolmogoroff A.N. (1937). K statisticheskoi teorii kristallizacii metallov. Izvestiya Akad. Nauk. SSSR Ser. Math..

[23] Evans U.R. (1945). The laws of expanding circles and spheres in relation to the lateral growth of surface films and the grainsizeof metals. Trans. Faraday Soc..

[24] Nakamura K., Watanabe T., Katayama K., Amano T. (1972). Some aspects of nonisothermal crystallization of polymers. I. Relationship between crystallization temperature, crystallinity and cooling conditions. J. Appl. Polym. Sci..

[25] Nakamura K., Katayama K., Amano T. (1973). Some aspects of nonisothermal crystallization of polymers. II. Consideration of the Isokinetic condition. J. Appl. Polym. Sci..

[26] Ozawa T. (1971). Kinetics of non-isothermal crystallization. Polymer.

[27] Billon N., Barq P., Haudin J. (1991). Modelling of the cooling of semi-crystalline polymers during their processing. Int. Polym. Proc..

[28] Haudin J.-M., Chenot J.-L. (2004). Numerical and physical modeling of polymer crystallization—Part I: Theoretical and numerical analysis. Int. Polym. Process.

[29] Schneider W., Koppl A., Berger J. (1998). Non-Isothermal crystallization of polymers. Int. Polym. Process..

[30] Kerner E. (1956). The Elastic and Thermo-elastic Properties of Composite Media. Proc. Phys. Soc. B.

[31] Van Dommelen J.A.W., Parks D.M., Boyce M.C., Brekelmans W.A.M., Baaijens F.P.T. (2003). Micromechanical modeling of the elasto-viscoplastic behavior of semi-crystalline polymers. J. Mech. Phys. Solids.

[32] Bedoui F., Diani J., Regnier G., Seiler W. (2006). Micromechanical modeling of isotropic elastic behavior of semicrystalline polymers. Acta Mater..

[33] Luo Y.-M., Detrez F., Chevalier L., Lu X., Roland S. (2023). Multiscale framework for estimation of elastic properties of poly ethylene terephthalate from the crystallization temperature. Mech. Mater..

[34] Monasse B., Haudin J.-M. (1986). Thermal dependence of nucleation and growth rate in polypropylene by non-isothermal calorimetry. Colloid. Polym. Sci..

[35] Billon N., Haudin J.-M. (1993). Determination of nucleation rate in polymers using isothermal crystallization experiments and computer simulation. Colloid. Polym. Sci..

[36] Billon N., Henaff V., Haudin J.-M. (2002). Transcrystallinity effects in high-density polyethylene. II. determination of kinetics parameters. J. Appl. Polym. Sci..

[37] Hoffman J.D. (1983). Regime III crystallization in melt-crystallized polymers: The variable cluster model of chain folding. Polymer.

[38] Hoffman J.D., Frolen L.J., Ross G.S., Lauritzen J.I. (1975). On the growth rate of spherulites and axiaites from the melt in polyethylene fractions: Regime I and regime II crystallization. J. Res. Natl. Bur. Stand. Sect. A Phys. Chem..

[39] Lachlan D.M. (1987). An equation for the conductivity of binary mixtures with anisotropic grain structures. J. Phys. C Solid. State Phys..

[40] Roy D., Audus D., Migler K. (2019). Rheology of crystallizing polymers: The role of spherulitic superstructures, gap height, and nucleation densities. J. Rheol..

[41] Schröter K., Hutcheson S.A., Shi X., Mandanici A., McKenna G.B. (2006). Dynamic shear modulus of glycerol: Corrections due to instrument compliance. J. Chem. Phys..

[42] Laukkanen O.V. (2017). Small-diameter parallel plate rheometry: A simple technique for measuring rheological properties of glass-forming liquids in shear. Rheol. Acta.

[43] Liu C.Y., Yao M., Garritano R.G., Franck A.J., Bailly C. (2011). Instrument compliance effects revisited: Linear viscoelastic measurements. Rheol. Acta.

